# Prognosis of aggressive adult T-cell leukemia/lymphoma with central nervous system infiltration and utility of CD7 versus CADM1 flowcytometric plots of cerebrospinal fluid

**DOI:** 10.1007/s00277-025-06186-4

**Published:** 2025-01-10

**Authors:** Koji Jimbo, Tomohiro Ishigaki, Masataka Sakashita, Shohei Andoh, Hirona Ichimura, Ayumu Ito, Kazuaki Yokoyama, Aki Sato, Takahiro Fukuda, Kaoru Uchimaru, Yasuhito Nannya

**Affiliations:** 1 Division of Hematopoietic Disease Control, The Institute of Medical Science, The University of Tokyo, Tokyo, Japan; 2 Department of Hematology/Oncology, Research Hospital, The Institute of Medical Science, The University of Tokyo, Tokyo, Japan; 3 Department of Laboratory Medicine, Research Hospital, The Institute of Medical Science, The University of Tokyo, Tokyo, Japan; 4 Department of Hematopoietic Stem Cell Transplantation, National Cancer Center Hospital, Tokyo, Japan; 5 Laboratory of Tumor Cell Biology, Department of Computational Biology and Medical Sciences, Graduate School of Frontier Sciences, The University of Tokyo, Tokyo, Japan

**Keywords:** Adult T-cell leukemia/lymphoma (ATL), Cerebrospinal fluid, Central nervous system infiltration, Flow cytometry

## Abstract

The prognosis of adult T-cell leukemia/lymphoma (ATL) with primary central nervous system (CNS) involvement has been unclear since the advent of new therapies. Recently, we have shown that flow cytometric CD7/CADM1 analysis of CD4 + cells (HAS-Flow) is useful to detect ATL cells that are not morphologically diagnosed as ATL cells. We investigated the role of CNS involvement in ATL using cytology and HAS-Flow by analyzing cerebrospinal fluid (CSF) from 73 aggressive ATL cases. Based on the findings in CSF, the study subjects were classified into CNS + (cytologically malignant, *n* = 18), CNS- (cytologically non-malignant and ATL cell population negative in HAS-Flow, *n* = 44), and CNS-Micro (cytologically non-malignant and ATL cell population positive in HAS-Flow, *n* = 11) groups. As expected, the CNS + group had a shorter overall survival than the CNS- groups (*P* < 0.001). However, the CNS-Micro group showed no adverse impact on overall survival compared to the CNS- group (*P* = 0.506), even without additional CNS-targeted treatments. HAS-Flow also demonstrated clinical utility in the diagnosis of CSF lesions in ATL patients with cerebral white matter lesions and in the detection of ATL cells on post-treatment CSF examination in patients with CNS involvement. Our study demonstrates that ATL with CNS involvement have a poor prognosis and that CSF HAS-Flow is useful to assist in the diagnosis of suspected CNS involvement and to detect ATL cells with high sensitivity after treatment.

**Supplementary Information:**

The online version contains supplementary material available at 10.1007/s00277-025-06186-4.

## Introduction

Adult T-cell leukemia/lymphoma (ATL) is a hematological malignancy with a poor prognosis that originates from human T-cell leukemia virus (HTLV)−1-infected T lymphocytes [1, 2]. Although primary central nervous system (CNS) lymphoma is known to have a poor prognosis [3], reports on the impact of CNS involvement on ATL outcomes are limited and show inconsistent results [4–6], leaving the prognostic significance of CNS involvement status at diagnosis undefined.

In addition to symptoms and imaging studies, cerebrospinal fluid (CSF) examination is important for diagnosing CNS involvement in lymphoma. Along with cell counts and cytology of CSF, the analysis of CSF cells for CADM1 and CD7 in CD4 + cells using flow cytometry (HAS-Flow) has been reported to be useful [7]. We recently reported that HAS-Flow analysis of peripheral blood is useful for prognostic estimation in aggressive ATL, demonstrating its ability to identify morphologically unidentified ATL cells in peripheral blood [8]. Therefore, we hypothesized that the effectiveness of HAS-Flow in detecting cryptic ATL cells could also be applied to CSF examinations for detecting CNS invasion. In addition, we explored the clinical impact of HAS-Flow-positive results.

## Methods

### Patients, Definitions, and Analyses

**Fig. 1:**
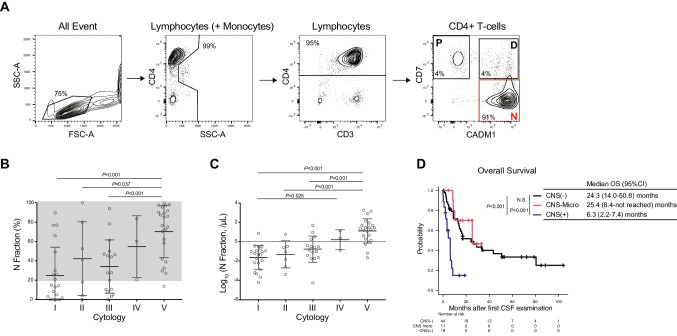
Effects of CADM1 versus CD7 flow cytometric plots and prognosis of aggressive ATL based on CNS infiltration status. **A** Representative flow cytometric plots using CSF samples from this study. **B**, **C** Proportion of the N fraction (%) (**B**) and absolute number of log-transformed N fraction (**C**) compared according to cytology results. **D** Overall survival of aggressive ATL patients compared by CNS infiltration status

Aggressive ATL patients treated at the Research Hospital, Institute of Medical Science, University of Tokyo, who had at least one CSF analysis between September 2014 and February 2024, were included. Data used in this study, including flow cytometric data, were extracted from medical records. The details of this observational study were published online, and consent of participants was obtained by an opt-out method [9]. ATL disease subtypes were classified according to the Shimoyama classification [10]. Pathological evaluation of CSF cells was performed in accordance with the standard Papanicolaou classification system [11] as follows: class I, normal; class II, benign atypia; class III, suggestive of malignancy; class IV, strongly suggestive of malignancy; and class V, consistent with malignancy. Class IV and V were considered malignant and denoted as Cyto + , while Classes I–III were categorized as Cyto-. Peripheral blood contamination of the CSF was determined by skilled pathologists. CSF examinations, including flow cytometry, were performed as part of the clinical assessment. Flow cytometric analyses were performed on CSF samples as previously reported for peripheral blood using a FACS Canto II (BD Biosciences), and the data were analyzed using FlowJo software (Tree Star) [8, 12]. Representative flow cytometric plots of CSF cells from this study are shown in Fig. 1A. Among CD4 + cells, HTLV-1 non-infected cells are represented as the P fraction (CD7 + /CADM1-), and among the HTLV-1 infected cell fractions (CADM1 + : D + N fractions), the N fraction (CD7-/CADM1 +) is shown to increase with clonal evolution [8, 12]. Samples with ≥ 20% and < 20% N fractions were assumed as HAS-Flow + and HAS-Flow-, respectively. This study was performed in accordance with the Declaration of Helsinki. The Institutional Review Board of the Institute of Medical Science, University of Tokyo, approved this study (2019–72–0217).

### Statistical analysis

Continuous variables were compared using Student’s t-test or the Kruskal–Wallis test. Variables were log-transformed when necessary to ensure a normal distribution. Categorical variables were compared using a chi-square test. Overall survival (OS) was analyzed based on the time since the first CSF examination and compared between groups by log-rank test. All statistical analyses were performed using GraphPad Prism software (version 7.0d; GraphPad Software), or EZR (version 1.55; Saitama Medical Center, Jichi Medical University) [13].

## Results

We analyzed 154 CSF samples from 73 patients with aggressive ATL. The median age at the time of the initial CSF examination was 65 years, with 58 patients (79%) having acute-type ATL (Table 1). CSF examination parameters by CSF cytology showed that the Class V group had significantly higher CSF cell counts, lactate dehydrogenase (LDH) values, and both the percentage and absolute number of ATL cell fractions (N fraction) by flow cytometry than the Class I–III group, which was not classified as malignant (Fig. 1B,C; S1A,B). We then evaluated 71 samples that underwent both CSF HAS-Flow and cytology analysis. CSF HAS-Flow was performed for screening at the time of initial CSF collection, to aid in the diagnosis of suspected CNS infiltration, and for follow-up of patients with CNS involvement. Of the 25 Cyto + samples, 24 were HAS-Flow + , whereas among the 46 Cyto- samples, 21 were HAS-Flow- (Fig. 1B,C). Thus, HAS-Flow had 96% sensitivity but only 46% specificity for cytological malignancy. This suggests the presence of CSF ATL cells that are not visually identified as well as cases with minimal CNS involvement among Cyto- patients. Based on the results of the initial CSF analysis, the patients were categorized into three groups: CSF + group (Cyto + without peripheral blood contamination), CNS-Micro group (Cyto- and HAS-Flow + without peripheral blood contamination), and CNS- group (all other cases). One patient, with an elevated CSF cell count (56/μL), a 94% N fraction on HAS-Flow, multiple cerebral white matter lesions on magnetic resonance imaging (MRI), and cytology classified as Class V on the second CSF examination was included in the CNS + group despite an initial CSF of Class III.

**Table 1:** Characteristics of patients, classified into 3 groups according to CNS involvement status

Patient	CNS(-), *n* = 44	CNS-Micro, *n* = 11	CNS( +), *n* = 18	*P* value	Total, *n* = 73
Median Age at First CSF Examination (range)	65 (49–82)	65 (41–73)	67.5 (41–76)	0.774	65 (41–82)
Sex, Male (%)	22 (50%)	5 (45%)	12 (67%)	0.416	39 (53%)
Disease Type at First Sample Collection				0.277	
Chronic (Unfavorable)	6 (14%)	3 (27%)	0 (0%)		9 (12%)
Lymphoma	3 (7%)	1 (9%)	2 (11%)		6 (8%)
Acute	35 (80%)	7 (64%)	16 (89%)		58 (79%)
Median Number of Samples per Patients (range)	1 (1–6)	2 (1–5)	3 (1–8)	< 0.001	2 (1–8)
Cases in which the first CSF sample was obtained after systemic therapy (%)	43 (98%)	11 (100%)	11 (61%)	< 0.001	65 (89%)
Cases with at least one time evaluation by flow cytometry (%)	15 (34%)	11 (100%)	16 (89%)	< 0.001	42 (58%)
Median Values at first CSF examination (range)					
Cells (/μL)	1 (0–113)	1 (1–11)	72 (5–3080)	< 0.001	2 (0–3080)
LDH, U/L	20 (11–33)	20 (14–39)	39 (21–327)	< 0.001	21.5 (11–327)
N fraction, %	3.5 (0–17.9)	34.6 (20.8–61.3)	86.8 (38.9–99.4)	< 0.001	33.4 (0–99.4)
Classifications of Cytology at first CSF Examination (%)				< 0.001	
I	28 (64%)	4 (36%)	0 (0%)		32 (44%)
II	9 (20%)	2 (18%)	0 (0%)		11 (15%)
III	6 (14%)	5 (45%)	1 (6%)		12 (16%)
IV	1 (2%)*	0 (0%)	0 (0%)		1 (1%)
V	0 (0%)	0 (0%)	17 (94%)		17 (29%)
Peripheral Blood Contamination to CSF at first CSF Examination (%)	7 (16%)	0 (0%)	0 (0%)		7 (10%)
Definite CNS lesions in the Overall Courses (%)	2 (5%)	1 (9%)	18 (100%)	< 0.001	21 (29%)
Therapy for CNS lesions (%)				0.050	
Intrathecal Injection of chemotherapy	44 (100%)	11 (100%)	18 (100%)		73 (100%)
Radiation	1 (2%)	0 (0%)	4 (22%)		5 (7%)
High Dose MTX + AraC	0 (0%)	0 (0%)	1 (6%)		1 (1%)
First Systemic Therapy for ATL (%)				0.387	
VCAP-AMP-VECP	24 (55%)	9 (82%)	8 (44%)		41 (56%)
CHOP like Regimen	9 (20%)	1 (9%)	2 (11%)		12 (16%)
Mogamulizumab ± Chemotherapy	8 (18%)	1 (9%)	4 (22%)		13 (18%)
Low Dose VP-16	1 (2%)	0 (0%)	0 (0%)		1 (1%)
High Dose MTX + AraC	0 (0%)	0 (0%)	1 (6%)		1 (1%)
Steroid only	1 (2%)	0 (0%)	0 (0%)		1 (1%)
Intrathecal Injection only	0 (0%)	0 (0%)	2 (11%)		2 (3%)
N/A (unknown initial treatment details)	1 (2%)	0 (0%)	1 (6%)		2 (3%)
Cases in which allo-HCT was performed (%)	26 (59%)	9 (82%)	5 (28%)	0.012	40 (54%)
Median Follow up time: days from first CSF collection (range)	515.5 (15–3171)	516 (139–1012)	131 (1–572)	< 0.001	403 (1–3171)

CNS, central nervous system; CSF, cerebrospinal fluid; LDH, lactate dehydrogenase; MTX, methotrexate; AraC cytarabine; VCAP, vincristine, cyclophosphamide, doxorubicin, and prednisolone; AMP, doxorubicin, ranimustine, and prednisolone; VECP, vindesine, etoposide, carboplatin, and prednisolone; CHOP, cyclophosphamide, vincristine, doxorubicin, and prednisolone; VP-16, etoposide; allo-HCT, allogeneic hematopoietic cell transplantation

^*^Peripheral blood contamination to CSF

Eleven percent of the patients in this cohort had their first CNS examination before initiation of systemic chemotherapy, most of whom were in the CNS + group (Table 1). The CSF cell counts and LDH values were not significantly different between the CNS- and CNS-Micro groups, whereas the ratio and absolute number of N fractions showed a stepwise increase in the CNS-, CNS-Micro, and CNS + groups (Figure S1C–F). All patients in this cohort, including the CNS- group, received intrathecal injection of chemotherapy (IT), and no further treatment was given to the CNS-Micro group for the control of CNS lesions (Table 1). Two patients in the CNS- group (5%) and one in the CNS-Micro group (9%) showed definite CNS involvement at the later follow-up (Table 1). The CNS + group had a significantly shorter OS compared with the CNS- and CNS-Micro groups, whereas there was no difference between the CNS- and CNS-Micro groups (Fig. 1D). Similar results were obtained when the analysis was limited to the acute-type ATL patients (Figure S2A). This suggests that CNS-Micro groups need no further CNS-targeted treatment in addition to prophylactic IT.

Allogeneic hematopoietic cell transplantation (allo-HCT) is a crucial treatment that contributes to improved prognosis for eligible patients with aggressive ATL [14]. Forty patients (54%) in this cohort as a whole underwent allo-HCT, while only 5 (28%) in the CNS + group underwent (Table 1). In this cohort as a whole, patients with allo-HCT had a better prognosis than non-transplant patients (Figure S2B). When comparing prognosis by CNS involvement status, only the CNS + group had a significantly worse prognosis than the other two groups as did the cohort as a whole, when limited to non-transplant cases (Figure S2C). However, there was no significant difference among the 3 groups in OS when limited to cases with allo-HCT (Figure S2D).

**Fig. 2:**
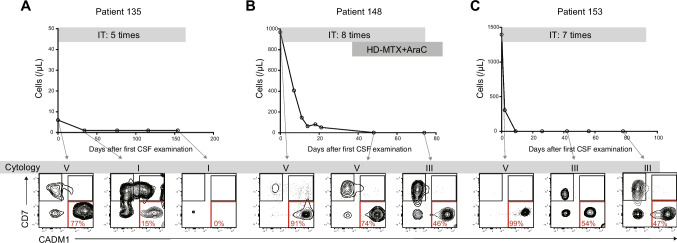
Usefulness of CADM1 versus CD7 flow cytometric plots in the follow-up of patients with CNS involvement. **A**–**C** CSF cell counts, cytology, and flow cytometric plots of the treatment course in three aggressive ATL patients with CNS involvement

We then reviewed three cases with CNS involvement that were evaluated for CSF by HAS-Flow repeatedly (Figs. 2A–C). Treatment for the CNS lesions reduced the cell counts to the normal range and changed malignant CSF cytology to the non-malignant category in all cases. However, the flow cytometric analyses showed that the N fraction disappeared in one case and remained in the other two cases. The two patients with a residual N fraction were subsequently treated with craniospinal irradiation followed by allo-HCT. One patient is alive without CNS lesion recurrence, while the other experienced CNS recurrence approximately 9 months after allo-HCT and died. These results suggest that HAS-Flow can detect ATL cells with high sensitivity and may be useful as a multifaceted evaluation of CNS lesions after treatment.

## Discussion

This is the first report showing that aggressive ATL patients with CNS infiltration at diagnosis have a poor prognosis, while simultaneously demonstrating that flow cytometric analysis of CSF is useful in detecting ATL cells.

Due to the rarity of the disease, the prognosis of CNS involvement in ATL was unclear. A report published more than 20 years ago showed that CNS involvement conferred no significant impact on OS [4]. The prognosis for the CNS + group in that report was 5.2 months from the time of CNS involvement detection, which is almost identical to this report (median OS 6.3 months). The treatment strategy for CNS lesions in ATL has not changed significantly over these 20 years, with IT or radiation being the mainstay [4, 6]. In contrast, the prognosis for ATL as a whole has improved with the advent of novel modalities, better allo-HCT outcomes, and improved supportive care, including infectious disease management [15]. This discrepancy in treatment progress between overall ATL and CNS-involved ATL is assumed to highlight the impact of CNS involvement on outcomes.

There are reports that the cases of CNS involvement with uncontrolled CNS lesions prior to allo-HCT are associated with poor prognosis, whereas the cases who achieved complete remission of CNS lesions are not associated with subsequent poor prognosis [6]. In this cohort, only a small number of patients in the CNS + group underwent allo-HCT. This indicates that there are a large number of patients in the CNS + group who did not achieve transplantation-eligible status because of the extremely poor prognosis. Actually, CNS + was not a significant prognostic factor only in cases with allo-HCT (Figure S2D). Therefore, this report supports the previous findings that CNS involvement does not confer poor prognosis when it is well-controlled prior to transplantation.

This report suggests that there is a group of patients with micro-CNS infiltration of ATL cells detected by flow cytometry alone, who would have been classified as CNS-negative without HAS-Flow. Unexpectedly, patients in this group did not have a poor prognosis, and many did not subsequently develop explicit CNS lesions. All patients in this cohort received routine prophylactic IT, suggesting that micro-CNS infiltration can be controlled by IT.

Although our results suggested that CNS-Micro (HAS-Flow + alone) has no prognostic impact on outcomes and requires no additional treatment, HAS-Flow analysis might be useful for identifying increased morphologically non-malignant CSF cells. In fact, we encountered a case with elevated CSF cell counts (56/μL) and a cerebral lesion on MRI. Cytology was Class III, but HAS-Flow was 96% positive, leading us to classify this case as CNS + and successfully treat the CNS lesion with IT.

It should be emphasized that the negligible impact of CNS-Micro on outcomes applies only to initial CSF samples before receiving CNS treatment; the role of CNS-Micro after treatment has not yet been determined. We had two patients whose explicit CNS + findings downgraded to CNS-Micro after CNS treatment. Both cases underwent allo-HCT following additional CNS radiotherapy, and one developed CNS recurrence after allo-HCT and died. These cases suggest that transient CNS-Micro findings after CNS treatment do not indicate favorable outcomes and should be treated with caution.

This study had several limitations. Firstly, it was a small, single-center, retrospective study. Large-scale, prospective studies are desirable. Secondly, HAS-Flow data were available for only 58% of the cases. There were cases in both the CNS + and CNS- groups where HAS-Flow was not performed (Table 1). A cohort with a larger number of analyzed cases would be desirable for investigating the utility of HAS-Flow. Thirdly, the prognosis of cases transitioning from CNS + to CNS-Micro after treatment has not been clarified. The accumulation of cases tracking the course of HAS-Flow in CNS + patients is desirable.

In conclusion, this study revealed that ATL cases with CNS involvement at diagnosis have a poor prognosis. HAS-Flow may be a potentially useful qualitative assessment of CSF cells in addition to cytology, but further validation is needed.

## Supplementary Information

Below is the link to the electronic supplementary material.

Supplementary file1 (PDF 548 KB)

Supplementary file2 (PDF 546 KB)

## Data Availability

No datasets were generated or analysed during the current study.
