# Supplementary material for: Prognosis of aggressive adult T-cell leukemia/lymphoma with central nervous system infiltration and utility of CD7 versus CADM1 flowcytometric plots of cerebrospinal fluid
Source: Ann Hematol. 2025 Jan 10;104(1):635–40. doi: 10.1007/s00277-025-06186-4 (PMC11868183; doi:10.1007/s00277-025-06186-4)
Supplement: Supplementary file 1 — Supplementary file1 (PDF 548 KB) [file 277_2025_6186_MOESM1_ESM.pdf]

Figure S1

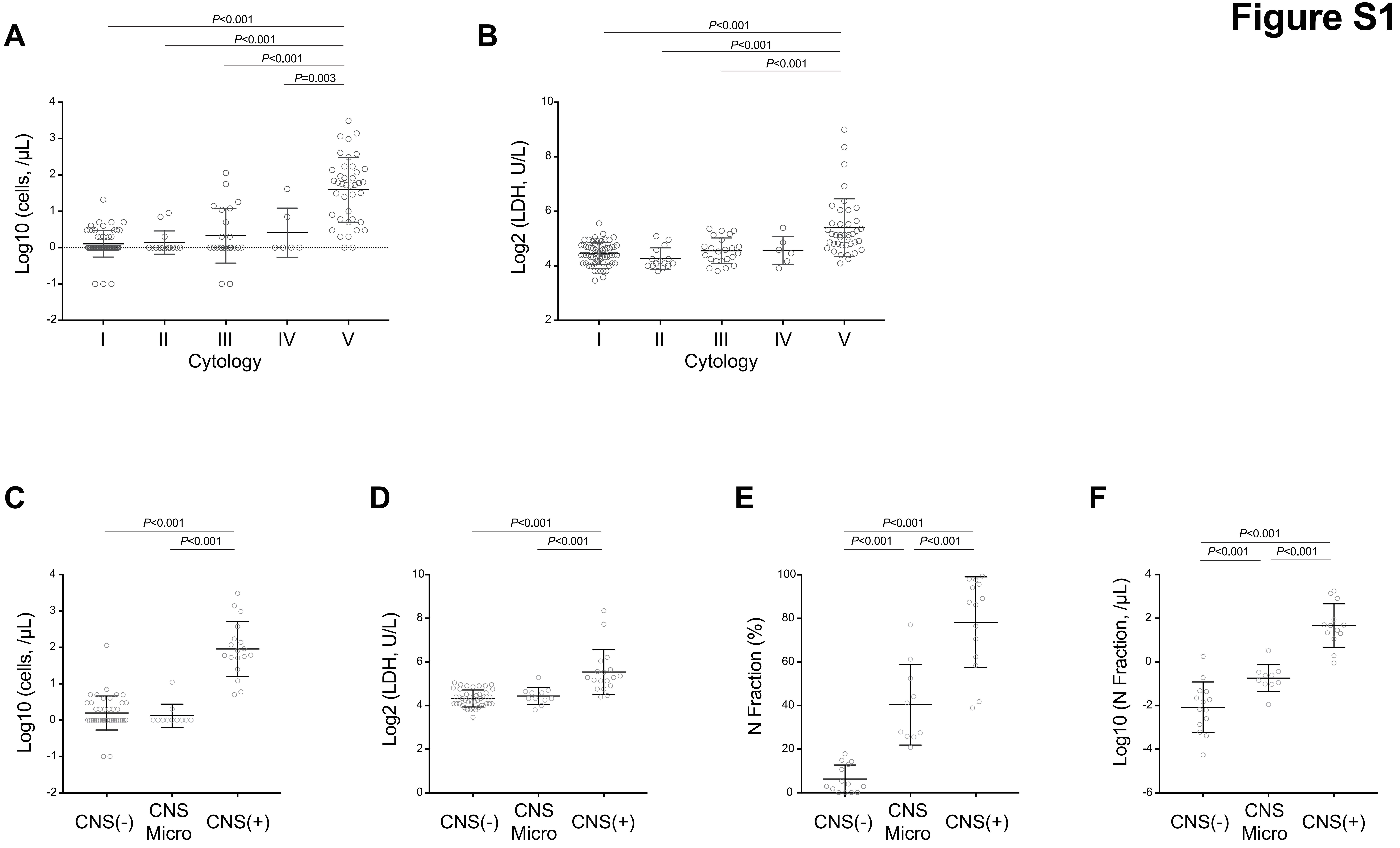

Supplementary Figure 1. Each parameters of CSF exmamination.
(A, B) Log-transformed CSF cell counts (A) and log-transformed lactate dehydrogenase (LDH) values (B), compared according to results of cytology.
(C-F) Log-transformed CSF cell counts (C), log-transformed LDH values (B), proportion of N fraction (%) (E), and absolute number of log-transformed N fraction (F), compared among the status of CNS infiltration.
