# Supplementary material for: Prognosis of aggressive adult T-cell leukemia/lymphoma with central nervous system infiltration and utility of CD7 versus CADM1 flowcytometric plots of cerebrospinal fluid
Source: Ann Hematol. 2025 Jan 10;104(1):635–40. doi: 10.1007/s00277-025-06186-4 (PMC11868183; doi:10.1007/s00277-025-06186-4)
Supplement: Supplementary file 2 — Supplementary file2 (PDF 546 KB) [file 277_2025_6186_MOESM2_ESM.pdf]

Figure S2

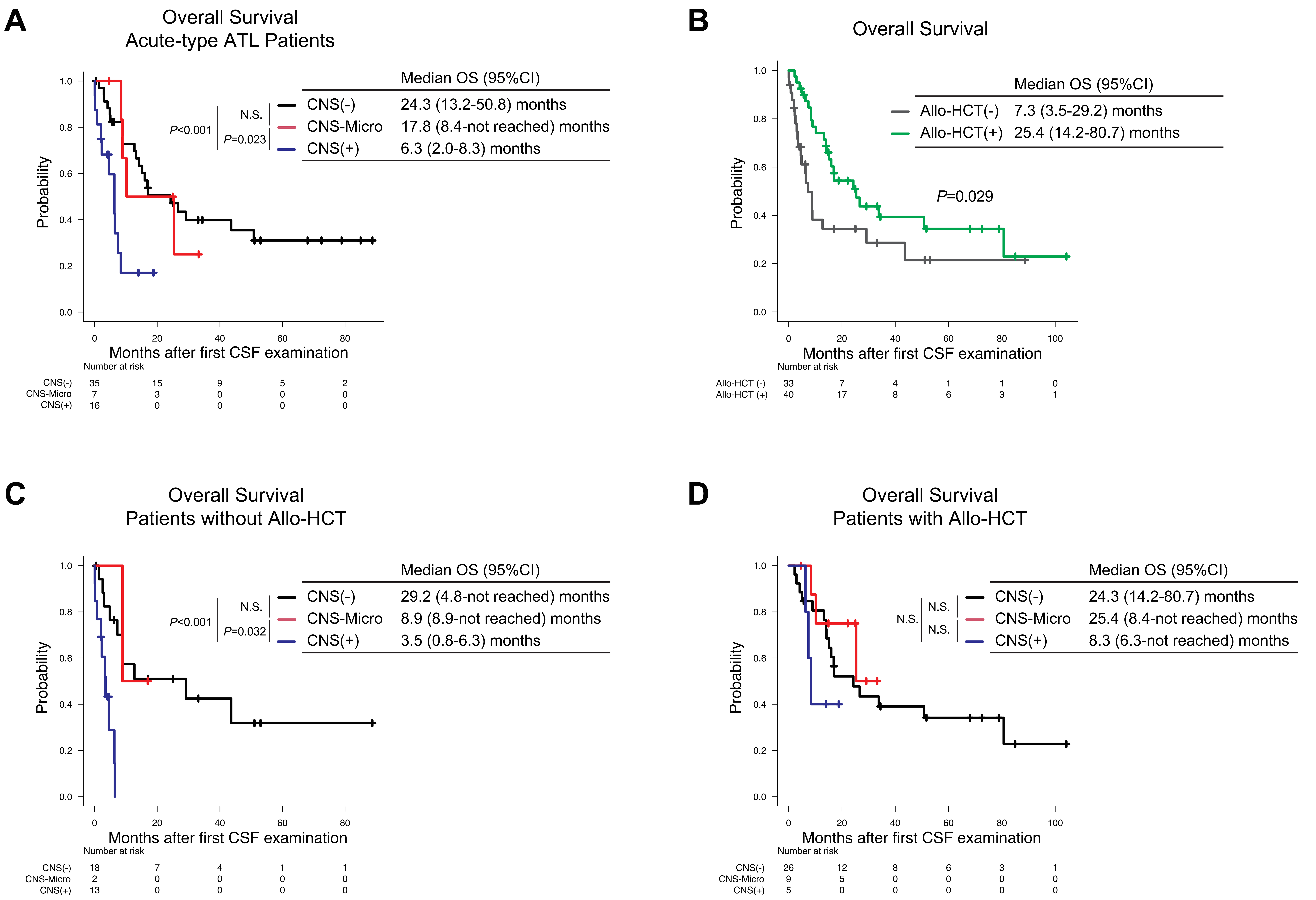

Supplementary Figure 2. Results of survival analysis in each subgroup.
(A) Overall survival of patients with acute-type ATL compared by CNS infiltration status (n=58).
(B) Overall survival of aggressive ATL patients compared with and without allo-HCT (n=73).
(C) Overall survival of aggressive ATL patients without allo-HCT compared by CNS infiltration status (n=33).
(D) Overall survival of aggressive ATL patients with allo-HCT compared by CNS infiltration status (n=40).
